# Supplementary material for: Identification of photoperiod-induced specific miRNAs in the adrenal glands of Sunite sheep (Ovis aries)
Source: Front Vet Sci. 2022 Jul 22;9:888207. doi: 10.3389/fvets.2022.888207 (PMC9354845; doi:10.3389/fvets.2022.888207)
Supplement: Supplementary file 1 [file Data_Sheet_1.ZIP › Supplementary file/miRDeep2 file.zip!/miRDeep2 file/Novel_Structure/SPLP42A1/Novel_469.pdf]

Provisional ID	:	NC_019484.2_32514
Score total	:	65.9
Score for star read(s)	:	3.9
Score for read counts	:	58.8
Score for mfe	:	1.7
Score for randfold	:	1.6
Score for cons. seed	:	
Total read count	:	127
Mature read count	:	118
Loop read count	:	0
Star read count	:	9

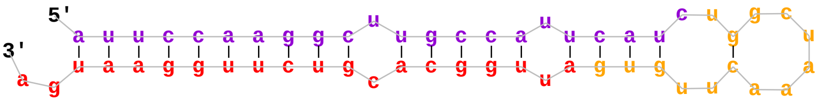

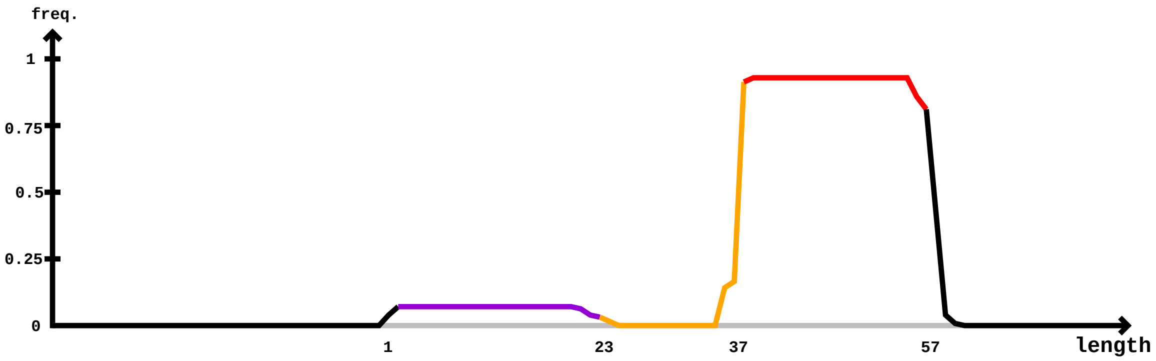

Star Mature

```
5'- uugccaccuucagcgugucugugugcuguaccccauuccaaggcuugccauucaucuggcuaaacuugugauuggcacgucuuggaaugaaguaacguacaacaaugaug   -3'    obs
    uugccaccuucagcgugucugugugcuguaccccauuccaaggcuugccauucaucuggcuaaacuugugauuggcacgucuuggaaugaaguaacguacaacaaugaug          exp
    ..((........))..((((((((((..(((..(((((((((((.(((((.((((..(......)..)))).))))).)))))))))))..)))..)))).)))..))).   reads     mm     sample
    .................................cauuccaaggcuugccauuc.........................................................   1         0      seq
    .................................cauuccaaggcuugccauuca........................................................   3         0      seq
    .................................cauuccaaggcuugccauucau.......................................................   1         0      seq
    ..................................auuccaaggcuugccauucauc......................................................   4         0      seq
    ....................................................................ugauuggcacgucuuggaau......................   6         0      seq
    ....................................................................ugauuggcacgucuuggaaug.....................   1         0      seq
    ....................................................................ugauuggcacgucuuggaauga....................   10        0      seq
    ....................................................................ugauuggcacgucuuggaaugaag..................   1         0      seq
    .....................................................................gauuggcacgucuuggaauga....................   2         0      seq
    .....................................................................gauuggcacgucuuggaaugaag..................   1         0      seq
    ......................................................................auuggcacgucuuggaau......................   3         0      seq
    ......................................................................auuggcacgucuuggaaug.....................   5         0      seq
    ......................................................................auuggcacgucuuggaauga....................   66        0      seq
    ......................................................................auuggcacgucuuggaaugaa...................   19        0      seq
    ......................................................................auuggcacgucuuggaaugaag..................   1         0      seq
    ......................................................................auuggcacgucuuggaaugaagu.................   1         0      seq
    .......................................................................uuggcacgucuuggaaugaa...................   1         0      seq
    .......................................................................uuggcacgucuuggaaugaag..................   1         0      seq
```
